# Enhanced osteoblastic differentiation of parietal bone in a novel murine model of mucopolysaccharidosis type II

**DOI:** 10.1016/j.ymgmr.2023.101021

**Published:** 2023-11-11

**Authors:** Narutoshi Yamazaki, Mari Ohira, Shuji Takada, Akira Ohtake, Masafumi Onodera, Mahito Nakanishi, Torayuki Okuyama, Ryuichi Mashima

**Affiliations:** a Department of Clinical Laboratory Medicine, National Center for Child Health and Development, 2-10-1 Okura, Setagaya-ku, Tokyo 157-8535, Japan; b Department of Clinical Genomics, Faculty of Medicine, Saitama Medical University, Saitama 350-0495, Japan; c Department of Systems BioMedicine, National Research Institute for Child Health and Development, 2-10-1 Okura, Setagaya-ku, Tokyo 157-8535, Japan; d Center for Intractable Diseases, Saitama Medical University Hospital, Saitama 350-0495, Japan; e Department of Human Genetics, National Research Institute for Child Health and Development, Tokyo, Japan; f TOKIWA-Bio Inc., 2-1-6 Sengen, Tsukuba City, Ibaraki 305-0047, Japan.

**Keywords:** Mucopolysaccharidosis II, Hunter syndrome, Iduronate-2-sulfatase, Bone deformity

## Abstract

Mucopolysaccharidosis type II (MPS II, OMIM 309900) is an X-linked disorder caused by a deficiency of lysosomal enzyme iduronate-2-sulfatase (IDS). The clinical manifestations of MPS II involve cognitive decline, bone deformity, and visceral disorders. These manifestations are closely associated with IDS enzyme activity, which catalyzes the stepwise degradation of heparan sulfate and dermatan sulfate. In this study, we established a novel *Ids*-deficient mice and further assessed the enzyme's physiological role. Using DNA sequencing, we found a genomic modification of the Ids genome, which involved the deletion of a 138-bp fragment spanning from intron 2 to exon 3, along with the insertion of an adenine at the 5′ end of exon 3 in the mutated allele. Consistent with previous data, our *Ids*-deficient mice showed an attenuated enzyme activity and an enhanced accumulation of glycosaminoglycans. Interestingly, we noticed a distinct enlargement of the calvarial bone in both neonatal and young adult mice. Our examination revealed that *Ids* deficiency led to an enhanced osteoblastogenesis in the parietal bone, a posterior part of the calvarial bone originating from the paraxial mesoderm and associated with an enhanced expression of osteoblastic makers, such as *Col1a* and *Runx2*. In sharp contrast, cell proliferation of the parietal bone in these mice appeared similar to that of wild-type controls. These results suggest that the deficiency of *Ids* could be involved in an augmented differentiation of calvarial bone, which is often noticed as an enlarged head circumference in MPS II-affected individuals.

## 1 Introduction

Mucopolysaccharidosis type II (MPS II, OMIM 309900) is an X-linked disorder characterized by an accumulation of glycosaminoglycan (GAG) in the body [1]. This disorder is caused by a deficiency of lysosomal enzyme iduronate-2-sulfatase (IDS), which catalyzes the degradation of heparan sulfate and dermatan sulfate. Disease manifestations of MPS II involve cognitive decline, bone deformity, and visceral disorders such as hepatosplenomegaly, umbilical hernia, upper airway obstruction, and cardiac dysfunctions [1,2]. Accumulating evidence of natural history indicated that MPS II may be clinically classified in two disease subtypes. A severe type of MPS II is associated with the involvement of the central nervous system (CNS) and becomes apparent in childhood. In contrast, an attenuated type of MPS II only shows visceral disorders. The result of a genotype-phenotype correlation study indicated that the attenuated type of MPS II is largely involved in missense mutations, whereas the severe type of MPS II is linked to recombination, large deletion, and frameshift [3]. Importantly, the percentage of the severe type of MPS II has been estimated as approximately 70%, which is closely linked to the high frequency of recombination of the *IDS-IDS2*, the latter being a pseudogene of the *IDS* gene which lacks enzyme activity. Enzyme replacement therapy is a currently used therapy that is effective for treating visceral manifestations [[4], [5], [6]]. A current challenge of developing novel therapies mainly focuses on improving CNS manifestation, such as cognitive decline. Based on this clinical demand, several therapeutic methods have been proposed. First, to deliver an enzyme agent more efficiently into the brain, intrathecal delivery of the enzyme has been developed [7,8]. Second, to more efficiently target the enzyme agent to the brain, recombinant enzyme agents that were fused to the anti-transferrin receptor antibody have been developed [[9], [10], [11], [12], [13]].

Abnormality of bone is often observed in MPS subtypes producing dermatan sulfate, such as MPS I, II, VI, and VII [2]. These bone manifestations are further divided into several phenotypes, such as an enlarged head circumference, short stature, dysostosis multiplex, and coarse facial features. Apart from such a variety of bone manifestations, the mechanism of bone development has been reconciled by two distinct mechanisms [14,15]. Endochondral ossification is a widely found osteogenesis mechanism in the long bones and is mediated by chondrogenesis. Intramembranous ossification, in contrast, forms an osteocyte from the osteoblast without chondrogenesis. The skeletal phenotype of MPS has been best studied by MPS VII mutant mice, because this animal model exhibits the most severe phenotype compared to other MPS disease models. In this case, the mechanism of short stature is, at least in part, linked to an impaired number and proliferation of chondrocyte associated with an impaired tyrosine phosphorylation of STAT3 [16]. In contrast, the mechanism of other bone phenotypes remains largely unclear.

The murine disease model has played an essential role for understanding the *in vivo* function of the gene and has been successfully applied to drug development. In the case of MPS II, a milestone study reported a detailed phenotype of *Ids*-deficient mice [17]. Subsequently, the offspring of this mouse line and others has been used for the extension study of phenotypes in many laboratories. In addition, these model mice were also used to test the efficacy of enzyme agents [7,12,13,18,19]. For the development of gene therapy, these mice were used for the efficacy and safety of vectors. For example, adeno-associated virus (AAV) is one widely used vector for gene therapy that has high infection efficiency. In the MPS II mouse model, some serotypes, such as AAV-2/5 [20], AAV-2/8 [21,22], and AAV9 [23,24] have been tested. Apart from this, the lentiviral vector is another therapeutic vector in humans that is frequently used for hematopoietic cells *ex vivo*. Furthermore, the possibility of the effect of gene correction in a mouse model has also been reported^,^ [25]. Overall, all these mice models consistently recapitulate the major phenotype in MPS II, such as an attenuated IDS enzyme activity and an enhanced accumulation of GAG. A recent study reported an enhanced intramaxillary width and zygomatic arch thickness as well as an enhanced bone volume/total tissue volume [25]. Noticeably, this bone phenotype was corrected by *ex vivo* lentiviral gene therapy, suggesting that the diseased osteoblast was treated with osteoclast through cross-correction [25]. In this study, we established an original murine model for MPS II and further characterized this model on the biochemical and histological basis. Through DNA sequencing, we detected a modification in the Ids genome, specifically involving the deletion of a 138-bp fragment extending from intron 2 to exon 3, and the insertion of an adenine at the 5′ end of exon 3 in the mutated allele. We particularly focused on the osteogenesis of the calvarial bones, such as the frontal bone and parietal bone.

## 2 Methods

### 2.1 Animal study

All experimental protocols have been approved by the institutional animal experiment committee of National Center for Child Health and Development (No. 18–3) and Saitama Medical University (No. 021 M129). Each method has been performed under authorized guidance and regulations. This study was carried out in compliance with the ARRIVE guidelines.

### 2.2 Establishing the *Ids*-deficient mouse model

A mutant mouse generation process involved the use of a gRNA with the following sequence: GTGAGGAAGGACACTCGACACTCGACTTGG, where the PAM was the sequence “TGG” at the end [26]. The sgRNA was synthesized using a CUGA7 gRNA synthesis kit from Nippon Gene Co., Ltd. (Toyama, Japan). A compound containing Cas9 protein (100 ng/μL), sgRNA (250 ng/μL), and ssODN (100 ng/μL) was injected into the nucleus and cytoplasm of *in vitro* fertilized oocytes obtained from C57BL/6 × DBA/2 F1 hybrid mice. The injected oocytes were cultured overnight and then transferred into the oviducts of pseudopregnant ICR females [27]. The resulting pups were identified using DNA sequencing.

### 2.3 Animal handling

C57BL/6 mice (CLEA Japan, Tokyo, Japan) were crossbred with the F0-generation mice. DNA sequencing was conducted to validate the genotypes of the resulting F1 mice. These F1 mice were subsequently intercrossed, giving rise to F2 offspring, which were utilized in subsequent experiments. The mice were maintained on a 12-h light and dark cycle from 8:00 AM to 8:00 PM and provided with standard chow *ad libitum* (CE-2, CLEA Japan, Tokyo, Japan). This study adheres to the ARRIVE guidelines (https://arriveguidelines.org). In this study, all the analyses of mice with the same genetic background involve comparing the wild-type control and *Ids-*deficient mice as littermates.

### 2.4 Genotyping PCR and DNA sequencing

A 1-mm tip of neonatal finger or a 3-mm tip of adult tail was excised and collected in a clean DNase-free PCR tube. Genomic DNA was prepared using Extract-N-Amp™ PCR kit (Sigma-Aldrich, St. Louis, MO), according to the manufacturer's instruction. After diluting the sample, PCR-mediated amplification was performed using ReadyMix™ Taq PCR Reaction Mix (Sigma-Aldrich) and ProFlex™ PCR System (Applied Biosystems, Waltham, MA). For further genome analysis, the PCR product from the wild-type control and *Ids*-deficient tissue was first separated by electrophoresis using 3% agarose gel. Then, a piece of gel containing the DNA was excised from gel. Finally, the target DNA was isolated using a Wizard genomic DNA purification kit (Promega, Madison, WI). For subsequent sample preparation, the reaction mixture was treated with an ExoSAP-IT Express PCR Product Cleanup kit (Applied Biosystems) and labeled with a BigDye Terminator v3.1 Cycle Sequencing Kit (Applied Biosystems). Finally, DNA sequencing was performed using a Genetic Analyzer 3130xl (Thermo Fisher Scientific, Waltham, MA).

### 2.5 Immunoblot analysis

An aliquot of cell lysate from tissue homogenate was electrophoresed on 10% Tris-HCl sodium dodecyl sulfate-polyacrylamide gel using Mini-PROTEAN ® TGX precast gel (4–20%, Bio-Rad, Hercules, CA) at 40 mA/gel. Then, the separated proteins were transferred onto a Trans-Blot ® Turbo PVDF membrane (0.2 μm, 7 × 8.5 cm mini format, Bio-Rad) at 400 mA/cassette for 7 min. Then, the membrane was treated with 3% skim milk (Snow Brand, Tokyo, Japan) in TBS containing 0.05% Tween-20 at room temperature for 30 min. Proteins on the membrane were reacted using a primary antibody from rabbit raised against mouse IDS protein (AF-2486, dilution 1:1000, R&D Systems, Minneapolis, MN) at room temperature for 1 h. After three washes using TBS containing 0.05% Tween-20, antigen on the PVDF membrane was reacted with horseradish peroxidase-conjugated IgG (GE Healthcare Life Sciences, Chicago, IL) in 1% skim milk (1:10,000). Finally, the target protein was visualized by ECL Western Blotting Detection Reagents (GE Healthcare Life Sciences). Chemiluminescence was detected using an Odyssey ® Fc Imaging System (LI-COR Biosciences, Lincoln, NE). For control loading, the PVDF membrane were reacted with β-Actin antibody (GTX 109639, dilution 1:1000, GeneTex, Irvine, CA).

### 2.6 GAG quantification

A piece of tissue (30–50 mg) was dissected and weighed. All tissues were homogenized in distilled water (1 mL) and reacted in lysis buffer (0.1 mg/mL proteinase K in 10 mmol/L Tris-HCl) at 56 °C for 16 h. Tissue debris was pelleted at 2000 ×*g* at 4 °C for 15 min, and supernatants were collected. The GAG concentration in tissue extract was quantified using a 1,9-dimethylmethylene blue-mediated colorimetric Blyscan Glycosaminoglycan Assay kit (Biocolor Life Science, Northern Ireland, UK) [27]. A standard curve was generated using chondroitin sulfate. An absorbance at 620 nm was recorded using a spectrophotometer FlexStation 3 (Molecular Devices, San Jose, CA) with a scan speed of 0.1 s/well.

### 2.7 Pathological examination

Organs were dissected, fixed in 10% neutral buffered formalin, and embedded in paraffin [28]. The bone tissues were decalcified in 10% EDTA solution (Muto Pure Chemicals Co., Ltd., Tokyo, Japan) at room temperature for 2 days. A section with 7-μm thickness was mounted on a glass slide. For a conventional histology examination, sections were stained with hematoxylin (FUJIFILM Wako Pure Chemical Corporation, Tokyo, Japan) and eosin (FUJIFILM Wako Pure Chemical Corporation). For immunohistochemistry, after deparaffinization with xylene, followed by rehydration through a graded series of alcohols, we further briefly rinsed sections in a 0.1 mol/L phosphate buffer solution. Next, sections were treated with 10 mM citrate at 95 °C for 15 min, 10% H_2_O_2_ at room temperature for 15 min, and 10% fetal bovine serum (FBS, GIBCO, Carlsbad, CA) at room temperature for 15 min. Then, the section was incubated with a primary antibody (Ki-67, ab 15,580, 1:1000, Abcam, Cambridge, UK; lysosomal-associated membrane protein-2, ab13524, 1:1000, Abcam) at 4 °C for 16 h. After three washes, the section was further reacted with biotin-labeled secondary antibody (Jackson ImmunoResearch, West Grove, PA) at room temperature for 1 h. After incubation, ABC Peroxidase Staining Kit (Thermo Fisher Scientific) was reacted for enhanced antigen detection. Finally, Peroxidase Stain DAB Kit (Nacalai Tesque, Kyoto, Japan) was used to detect labeled antigen. All histological sections were examined using BZ-X710 (Keyence, Osaka, Japan).

### 2.8 IDS enzyme activity

Enzyme activity was examined as previously described [29]. In brief, a 3-mm punch of dried blood spot was incubated with substrate at 37 °C for 20 h (PerkinElmer). After the enzyme reaction was completed, it was halted by using a mixture of methanol and ethyl acetate.The reaction products were then extracted into ethyl acetate, facilitated by a 96-well plate. The resultant products were then reconstituted using a mixture of acetonitrile and water containing 0.2% formic acid [27]. Enzyme activity was measured using liquid chromatography-tandem mass spectrometry.

### 2.9 Tissue harvesting and osteoblast isolation

Frontal osteoblasts and parietal osteoblasts were isolated from the skull of mice at postnatal day (P) 0–2 [30]. Then, pericranium and dura mater were dissected from the skull and the cranial suture was also removed. Subsequently, frontal bone and parietal bone were minced in a separate 1.5-mL microtube. Small pieces of bone were then digested with 0.1% Collagenase A (Nacalai Tesque, Kyoto, Japan) in serum-free α-MEM six times at 37 °C for 10 min. We discarded the first two digestions; the latter four digestions were pooled together. All of the collected digestions were neutralized with an equal volume of growth media containing α-MEM supplemented with 10% FBS, 1% penicillin, and streptomycin (GIBCO). Osteoblasts isolated from frontal bone and parietal bone were plated in 100-mm tissue culture dishes (Corning Incorporated, New York, NY) and incubated at 37 °C in a 5% CO_2_ incubator. The medium was changed twice a week. Cells at passage 1 and 2 were used for all experiments.

### 2.10 Osteogenic differentiation

Osteoblasts isolated from frontal bone and parietal bone were plated on a 6-well-plate (1.0–2.0 × 10^5^ cells/well). When cells were grown in sub-confluence, they were incubated with an osteogenic differentiation medium, containing α-MEM supplemented with 10% FBS, 1% penicillin/streptomycin, 10 μM glycerol β-phosphate (Nacalai Tesque), and 0.25 μM ascorbic acid (Nacalai Tesque). The medium was changed twice a week. Mineralization of extracellular matrix was assessed by Alizarin red staining at week three of differentiation.

### 2.11 RNA isolation, cDNA synthesis, and quantitative RT-PCR

Total RNA was isolated from the tissues and cells by Sepasol®-RNA I Super G (Nacalai Tesque). The concentration of isolated RNA was determined spectrophotometrically using a NanoDrop2000 (Thermo Fisher Scientific). The cDNA was synthesized using a GeneAce cDNA synthesis Kit (Nippon gene, Tokyo, Japan) at 42 °C in the presence of oligo (dT)_15_ primer (Takara Bio, Tokyo, Japan) according to the manufacturer's instructions. Then, PCR amplification was carried out with THUNDERBIRD® SYBR qPCR Mix (Toyobo, Tokyo, Japan) using cDNA as a template. The PCR primers were described in Supplementary Table S1. Level of gene expression was normalized to *Gapdh* and presented as fold increase compared to the wild-type control.

### 2.12 Cell proliferation assay

Cell proliferation was assayed by CCK8 (Dojindo, Kumamoto, Japan) according to the manufacturer's instruction. In brief, differentiated cells were plated on a 96-well plate at 5.0 × 10^3^ cells/well. Then, the plate was incubated with CCK8 working solution at 37 °C for 4 h. Finally, the proliferation of cells was assessed by absorbance at 450 nm using a spectrophotometer Wallac 1420 ARVO sx (PerkinElmer, Waltham, MA). All assays were performed in triplicate.

### 2.13 Measurement of skull width

The skull width was measured using a digital caliper (Shinwa Sokutei, Niigata, Japan).

### 2.14 HEXB enzyme activity

Enzyme activity of HEXB was measured with 1 mmol/L 4-methylumbelliferone-labeled substrate of HEXB in 100 mmol/L acetate buffer (pH 5.0, 50 μL) in a 96-well plate at 37 °C for 20 h [24]. Then, the reaction was stopped by adding the same volume of 5 mmol/L potassium hydroxide to each well. An absorbance at 460 nm was recorded using a spectrophotometer Wallac 1420 ARVO sx (PerkinElmer). A standard curve was generated using unlabeled 4-methylumbelliferone. All assays were performed in duplicate.

### 2.15 Protein assay

An aliquot (25 μL) of tissue homogenate was mixed with bicinchoninic acid protein assay reagent (200 μL) (Nacalai Tesque) and incubated at 37 °C for 30 min. After the reaction, absorbance at 562 nm was recorded using a spectrophotometer FlexStation 3 (Molecular Devices). BSA was used as a standard.

### 2.16 Osteoblastic GAG content

We established modified cell lines derived from baby hamster kidney cells (BHK-21 cells) that consistently produce human IDS. In the culture supernatant, we measured IDS activity to be 2 μmol/h/L [33]. Separately, *Ids*-deficient parietal bone-derived osteoblasts were cultured and differentiated in a 96-well plate at 37 °C under 5% CO2, following the procedure described above. Following cell culture, we added an aliquot of this IDS-enriched culture supernatant and incubated the cells for an additional 24 or 48 h. After incubation, cellular proteins were solubilized using a 100 μL solution of CelLytic M (Sigma-Aldrich, St Louis, MO). Finally, the concentration of cellular GAGs in the lysate (50 μL) was determined using a 1,9-dimethylmethylene blue-mediated colorimetric Blyscan Glycosaminoglycan Assay kit as described.

### 2.17 Statistical analysis

Statistical significance of the difference between mean values are examined by Student's test. *P* value <0.05 was considered statistically significant. Data were expressed as mean ± standard error of mean.

## 3 Results

### 3.1 Generation of *Ids*-deficient mice

**Fig. 1:**
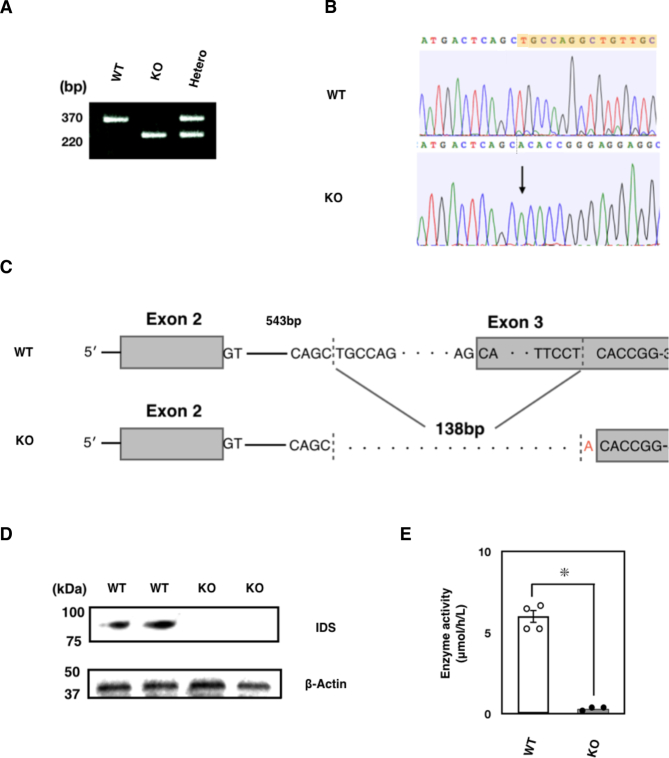
Genotyping of *Ids*-deficient mice. (A) PCR amplification of genomic DNA isolated from mouse tail biopsies. A single band corresponding to the amplicon of wild-type *Ids* allele with 370-bp and that of *Ids*-deficient allele with 220-bp were shown. Sequence of primers was summarized in Supplementary Table S1. (B) Sequencing electropherogram of wild-type and *Ids*-deficient allele. The existing sequence in the wild-type allele was indicated by the color yellow. (C) The schematic representation of mutated *Ids* genome structure showed a 138-bp deletion and an A insertion spanning from intron 2 to exon 3. (D) Immunoblot analysis of mouse liver extracts from wild-type and *Ids*-deficient mice using antibodies against IDS. A 90-kDa band of IDS protein was observed in the wild-type homogenate. Under these conditions, no band of IDS protein was detected at the same position in the Ids-deficient sample.(E) The IDS enzyme activity measured using a liquid chromatography-tandem mass spectrometry. The dried blood spot prepared from *Ids*-deficient mice (*n* = 3) showed reduced IDS enzyme activity relative to wild-type controls (*n* = 4).

**Supplementary Fig. 1:**
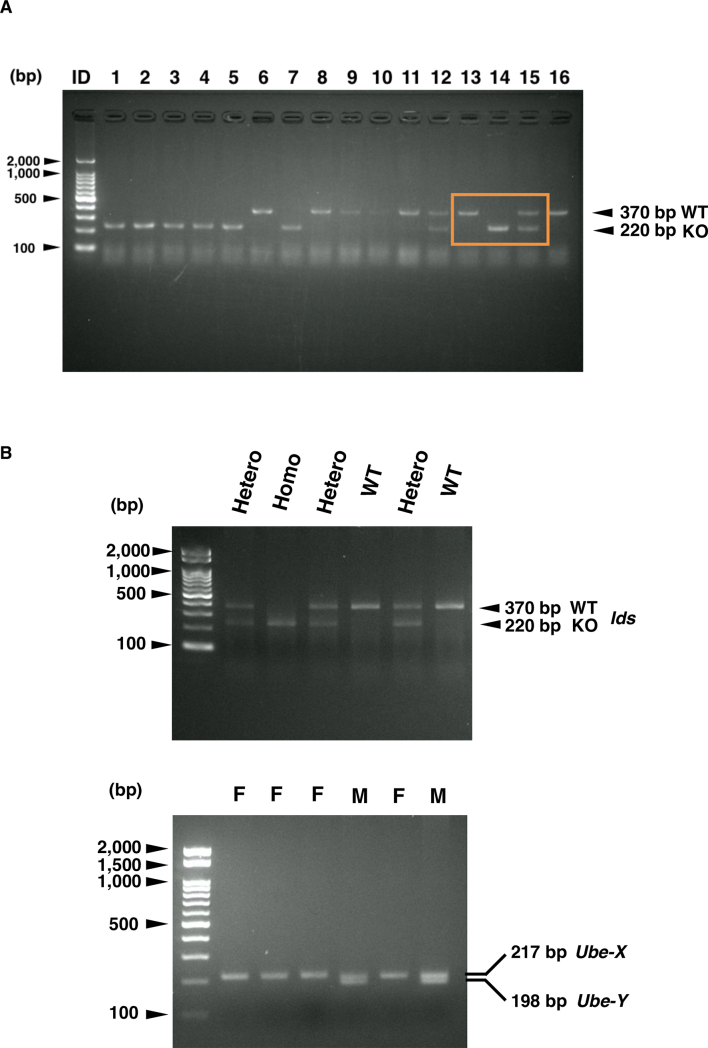
Genotyping of *Ids*-deficient mice.

**Supplementary Fig. 2:**
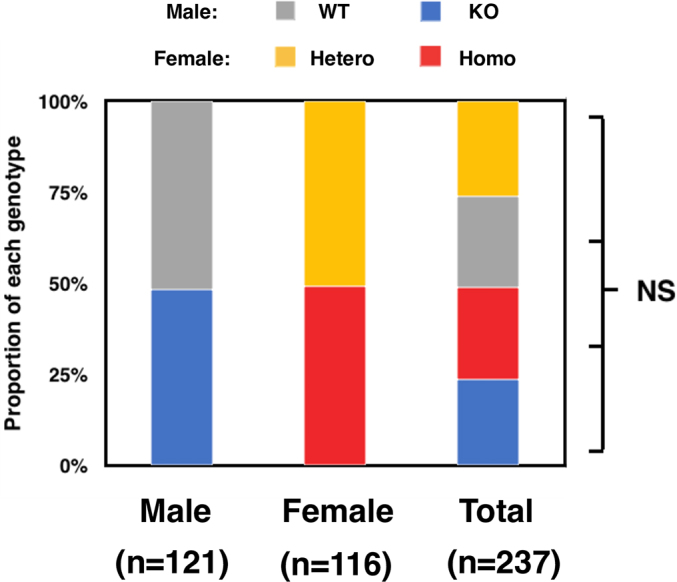
Ratio of mutant animals as determined by genotyping.

**Supplementary Fig. 3:**
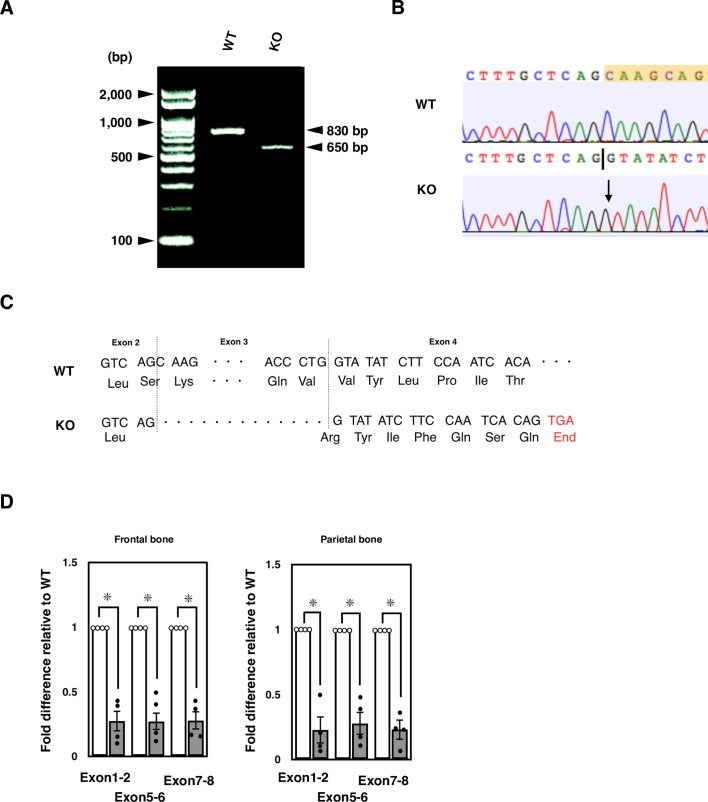
Nucleotide sequence and expression of matured mRNA in *Ids*-deficient mice used in this study.

Previous studies have established several lines of *Ids*-deficient mice with homologous recombination. In this study, we originally established novel *Ids*-deficient mice. In these animals, the mutant mice have shorter genomes as was detected as a single PCR product with approximately 220 bp in size compared to wild-type controls which have an amplicon with 370 bp (Fig. 1A and Supplementary Fig. S1). This mutated allele was passed down to offspring with Mendelian manner in both male and female (Supplementary Fig. S2). Using DNA sequencing, we found a genomic modification of the *Ids* genome spanning from intron 2 to exon 3 (Fig. 1B). More specifically, a 138-bp fragment of the *Ids* genome was missing and an adenine was additionally inserted at the 5′ end of exon 3 in the mutated allele (Fig. 1C). Consistently, these mice lacked IDS enzyme activity when a dried blood spot was used as the source of an enzyme (Fig. 1D). Note that a detailed nucleic sequence analysis of cDNA revealed that the exon 3 was absent in mRNA with the emergence of a termination codon, TGA, in *Ids*-deficient mRNA sequence (Supplementary Fig. S3). By quantitative RT-PCR, we also found that the expression of exons 1–2, 5–6, and 7–8 of the *Ids* gene was similarly impaired in the mRNA of both the frontal bone and parietal bone, demonstrating that an instability of mRNA in *Ids*-deficient mice could also be involved in the pathogenesis.

### 3.2 Visceral phenotype of *Ids*-deficient mice

**Fig. 2:**
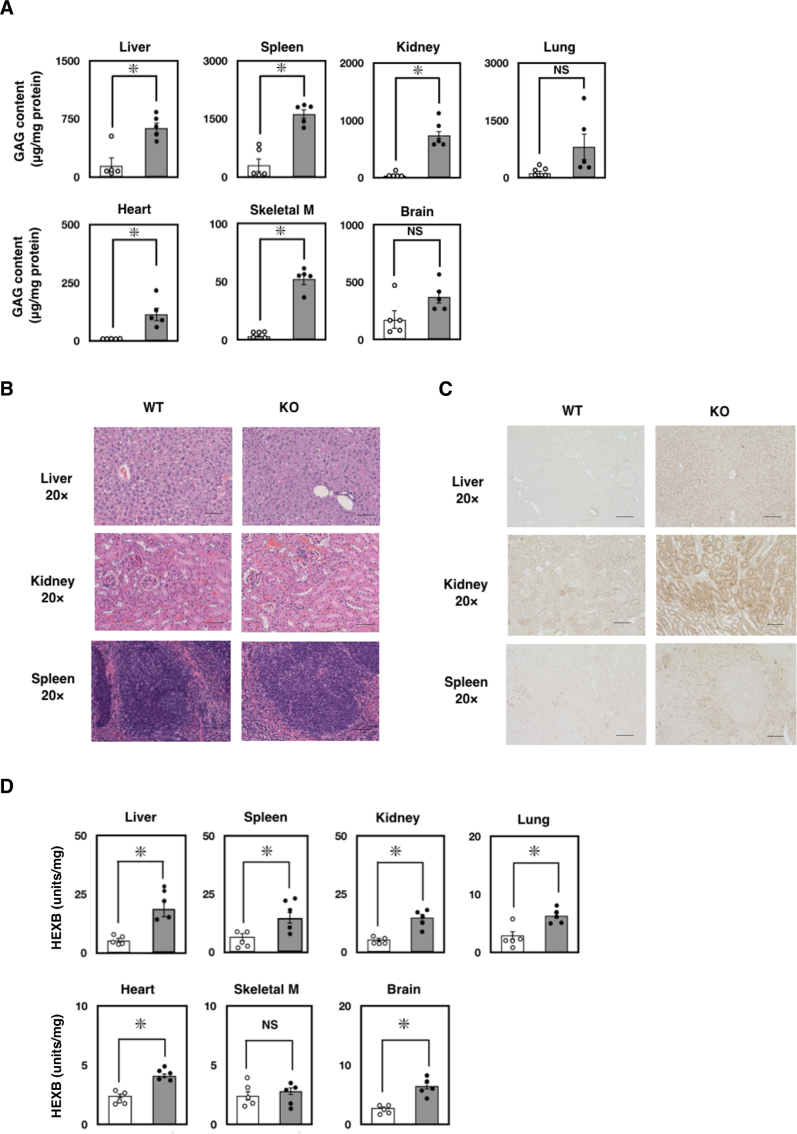
Visceral phenotype of *Ids*-deficient mice. (A) The GAG concentration in the organ. At 13 weeks of age, the GAG concentration in the liver, spleen, kidney, lung, heart, skeletal muscle, and brain was quantified using a 1,9-dimethylmethylene blue-mediated colorimetric method. The GAG concentration was expressed as micrograms per milligram protein. (B) Pathological appearance of *Ids*-deficient mice at 8 weeks of age. A formalin-fixed specimen from the liver, kidney, and spleen was treated with graded concentration of ethanol, xylene, and embedded in paraffin. A 7-μm section was stained with hematoxylin and eosin. Scale bar indicates 100 μm. (C) The LAMP2-staining of *Ids*-deficient mice. A 7-μm section was stained with anti-LAMP2 antibody (Abcam). Scale bar indicates 100 μm. (D) Levels of HEXB enzyme activity in the tissue at 13 weeks of age. Tissue homogenate was prepared in water and the enzyme activity and protein concentration in the aliquot was quantified. Enzyme activity of HEXB was quantified using a 4-methylumbelliferone-mediated fluorometric assay. Protein concentration was determined by BCA assay using BSA as a standard.

**Supplementary Fig. 4:**
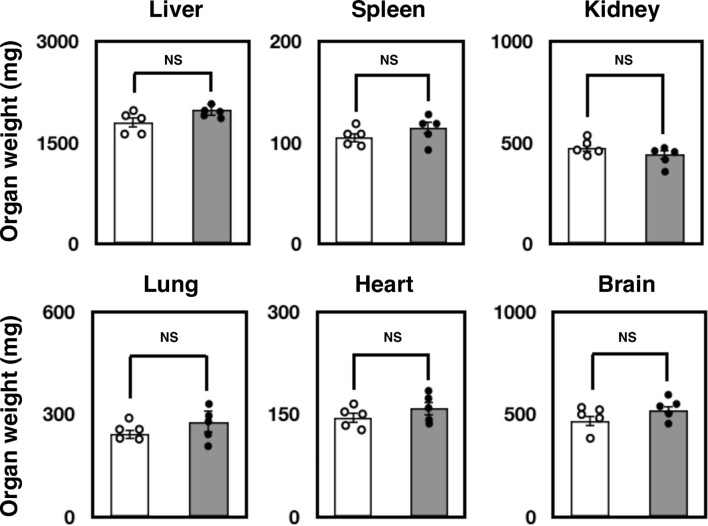
The weight of organ was examined at 13 weeks of age

The IDS enzyme plays a key role in the lysosomal degradation of GAG in the body. Thus, we reasoned a marked elevation of GAG in our newly established *Ids*-deficient mice. Consistently, when examining mice at 13 weeks of age, we found a significant elevation of GAG in the liver (4-fold), spleen (5-fold), kidney (22-fold), lung (8-fold), heart (13-fold), skeletal muscle (17-fold), and brain (3-fold) compared to the wild-type control (Fig. 2A). When we examined, there appeared to be almost no alteration in the pathology of organs examined by hematoxylin and eosin staining as well as in the tissue weight at 13 weeks of age (Fig. 2B and Supplementary Fig. S4). In contrast, a marked staining of LAMP2, an established marker for lysosomal storage disorder (LSD) was increased (Fig. 2C). Previous studies have indicated that, in the *Ids*-deficient mice, there is a marked elevation of β-hexosaminidase (HEXB)—a lysosomal enzyme responsible for Sandhoff disease—in the body. In fact, we also found an elevation of HEXB enzyme activity in the organs of *Ids*-deficient mice when mice were examined at 13 weeks of age (Fig. 2D).

### 3.3 Skeletal phenotype of *Ids*-deficient mice

**Fig. 3:**
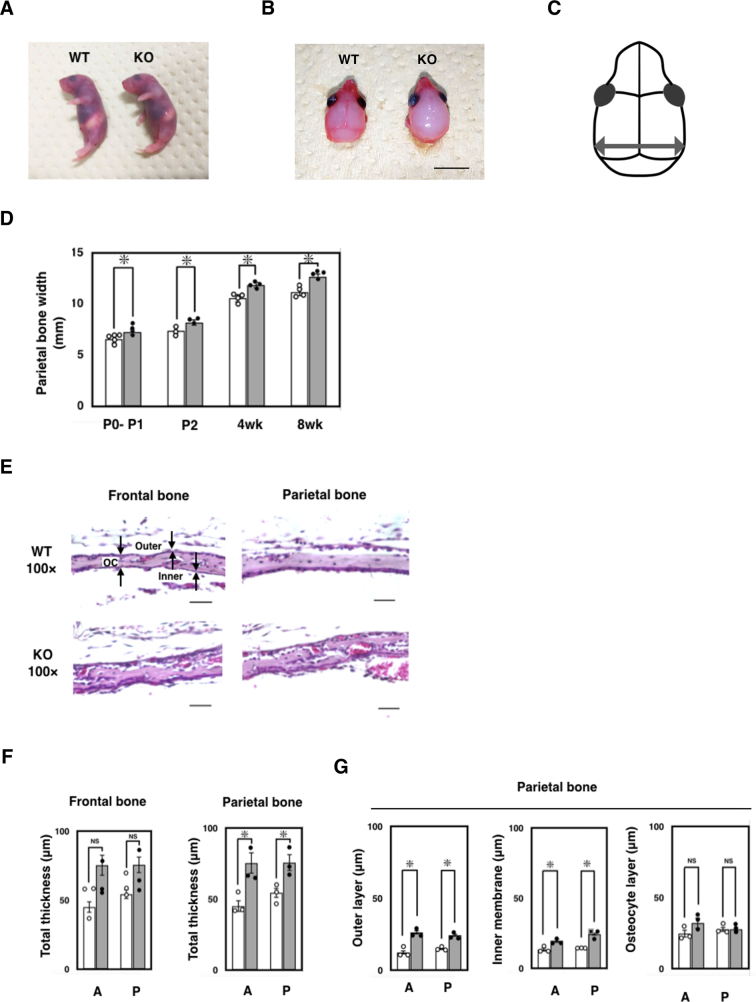
Skeletal phenotype of *Ids*-deficient mice. (A) Gross appearance of P0 mice. A picture of wild-type (left) and *Ids*-deficient mice (right) mouse was shown. (B) Representative picture of the skull of newborn mice (P0). The scalp was removed before being photographed. An enlarged skull in the *Ids*-deficient mice was noticed (Right, KO). (C) Schematic representation of the mouse skull. An arrow represents the maximum width of the skull. (D) Changes in the skull width in *Ids*-deficient mice. After the removal of the scalp, the width of the exposed skull was measured using a digital caliper. The number of animals examined at each time point was *n* = 3–5. (E) Representative photograph of sagittal section of the skull at P0–1/P2. Calvarial bone was isolated, formalin-fixed, and decalcified using 10% EDTA. Paraffin-embedded tissue was sectioned at 7-μm in thickness. Sections were stained using hematoxylin and eosin. Scale bar = 20 μm. The outer and inner layers of osteoblast (Outer and Inner) and the layer of osteocyte (OC) were shown with arrows. (F) Total thickness of frontal bone and parietal bone. The sum of thickness of outer and inner layers of osteoblast and that of osteocyte were expressed as total thickness. Both anterior (A) and posterior (P) sites of frontal bone and parietal bone were separately examined (wild-type, *n* = 3; *Ids*-deficient, *n* = 3). (G) Changes in the thickness of an outer and inner layer of osteoblast and a layer of osteocyte in P0-P1 (or P2) mice. Note that open and gray bar represents the mean value of wild-type controls (*n* = 3) and *Ids*-deficient animals (*n* = 3), respectively.

**Supplementary Fig. 5:**
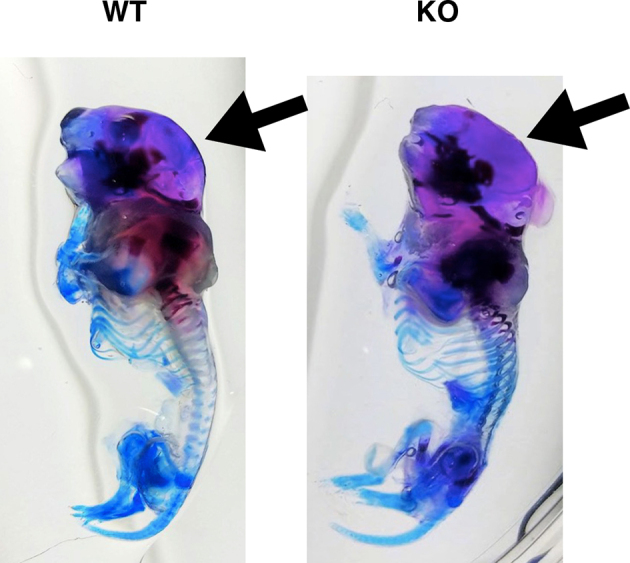
Whole-mount Alizarin Red S/Alcian Blue staining.

MPS II has three major phenotypes, such as CNS involvement, skeletal manifestation, and visceral disorders. In this study, among these phenotypes, we were particularly interested in skeletal manifestation, because effective treatment is limited. In P0–1/P2, we hardly recognized an altered appearance of the whole body in *Ids*-deficient mice (Fig. 3A). Thus, we further decided to explore the skeletal alteration in the calvarial bone in *Ids*-deficient mice, including the parietal bone and frontal bone (Fig. 3B). Intriguingly, we found an enlarged calvarial bone in *Ids*-deficient mice, but not in the wild-type controls. Based on this observation, we defined the width of the parietal bone (Fig. 3C) and measured it using a digital caliper (Fig. 3D). In fact, the width of the parietal bone was approximately 10% increased across P0-P1 to 8 weeks of age. Consistently, these *Ids*-deficient animals showed an enhanced development of the parietal bone as demonstrated in red by the whole mount staining (Supplementary Fig. S5).

**Supplementary Fig. 6:**
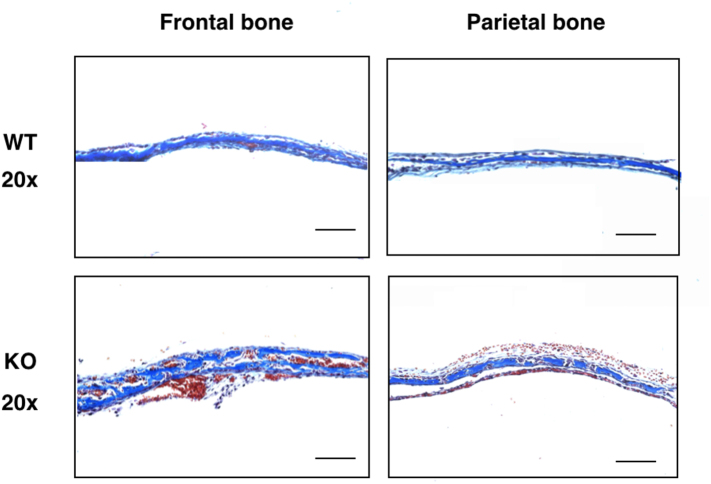
Masson’s trichrome staining of cranial bone.

Then, we examined pathological changes of the calvarial bone. Since this developmental enhancement was markedly enhanced in the parietal bone rather than the frontal bone (Fig. 3B), we further examined the pathological changes more carefully. It is known that the calvarial bone consists of two layers of osteoblast and one layer of osteocyte (Fig. 3E). These layers were readily distinguished by hematoxylin and eosin staining. Thus, we defined these layers and measured their thickness. In both the frontal bone and parietal bone, although there was an increasing trend in total thickness (*i.e.*, outer osteoblast layer + osteocyte layer + inner osteoblast layer), only the total thickness of the parietal bone, but not the frontal bone, was significantly increased in *Ids*-deficient mice (Fig. 3F). This result was further ensured when the anterior (A) and posterior (P) sites of each frontal bone and parietal bone were examined. Note that the thickness of the osteocyte layer, as examined by Masson's Trichrome staining, remained unaltered (Supplementary Fig. S6). Thus, to identify which layer became thicker in *Ids*-deficient mice, we measured their thickness individually. We found an enhanced thickening of both outer and inner layers of the osteoblast at both the anterior and posterior sites in the parietal bone, but not of an osteocyte layer (Fig. 3G). These results indicated that the deficiency of *Ids* might be linked to an enhanced osteoblastogenesis *in vivo*.

**Fig. 4:**
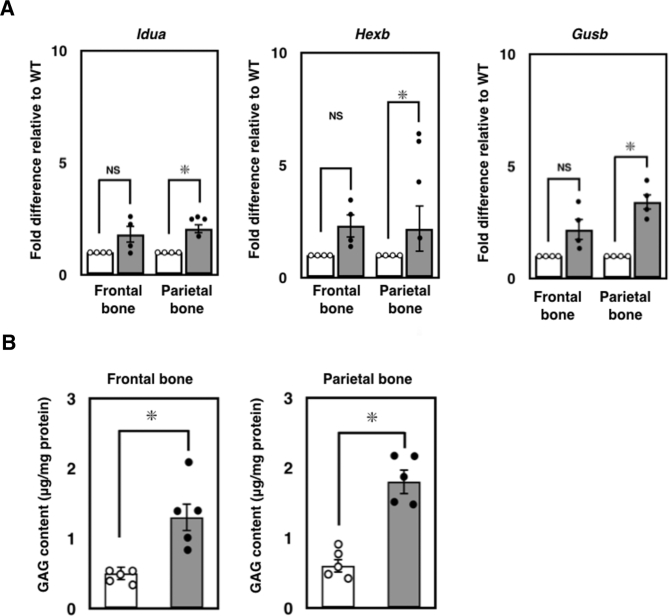
Gene expression changes in lysosomal storage disorder-related genes and GAG concentration in frontal and parietal bones in *Ids*-deficient mice. (A) Changes in the expression of lysosomal storage disorder-related genes in frontal bone and parietal bone. The expression of *Idua*, *Hexb*, and *Gusb* in P0–1/P2 was examined by quantitative RT-PCR. *Gapdh* was used as a control. (B) The GAG concentration in the frontal bone and parietal bone. Both frontal bone and parietal bone were surgically isolated and homogenized in distilled water. Then, the homogenate was treated with 0.1 mg/mL Proteinase K in 10 mM Tris-HCl buffer at 56 °C for 16 h. The concentration of solubilized GAG was quantified using a 1,9-dimethylmethylene blue-mediated colorimetric method. The GAG content was expressed as μg/mg protein.

To further gain insight into the molecular mechanisms involved in this skeletal phenotype in the parietal bone in *Ids*-deficient mice, we performed an experiment to understand whether the *Ids*-deficiency leads to another unique alteration of gene expression in the parietal bone and frontal bone. Thus, we surgically isolated the parietal bone and frontal bone, extracted total RNA, reverse transcribed it. To ensure the measurement of gene expression, we performed a quantitative RT-PCR. In fact, we were also able to detect an enhanced expression of *Idua, Hexb,* and *Gusb* by quantitative RT-PCR in the parietal bone, but not the frontal bone (Fig. 4A). This demonstrated that the deficiency of *Ids* led to an altered expression of the LSD-related gene in the parietal bone, and to a lesser extent, in the frontal bone through a common mechanism found in other organs as evaluated by the expression of the HEXB enzyme (Fig. 2D). Consistent with this, we also found an elevation of GAG in both the parietal bone and frontal bone (Fig. 4B). This further supported the notion that the *Ids* deficiency in our animal model caused an altered gene expression in calvarial bones such as the parietal bone and frontal bone.

**Fig. 5:**
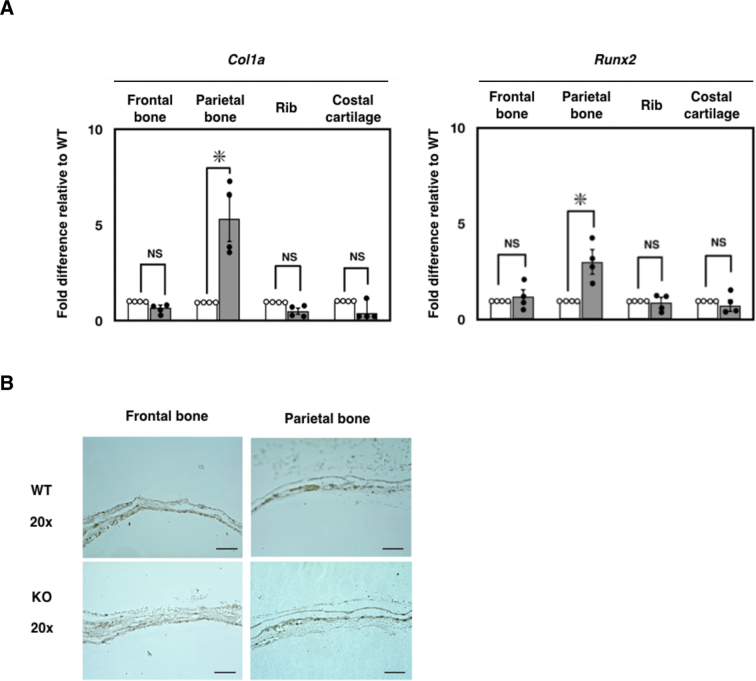
Enhanced osteoblastic differentiation of parietal bone in *Ids*-deficient mice. (A) Changes in expressing osteoblast-related genes in frontal bone and parietal bone. Total RNA was extracted from frontal bone, parietal bone, ribs, and costal cartilage isolated from P0–2 mice. Measurement of mRNA expression of *Col1a* and *Runx2* was performed with quantitative RT-PCR. Expression level was normalized to *Gapdh*, and the data were plotted relative to the values of wild-type for each gene. An asterisk (*) represents a significant difference between groups (*P* < 0.05). Values show mean ± SEM (*n* = 4). (B) Normal expression of Ki-67 in frontal bone and parietal bone in *Ids*-deficient mice by immunohistochemistry. Expression of Ki-67 was enhanced in the osteoblast, but not in the osteocyte, in these mice. Scale bar = 100 μm.

**Supplementary Fig. 7:**
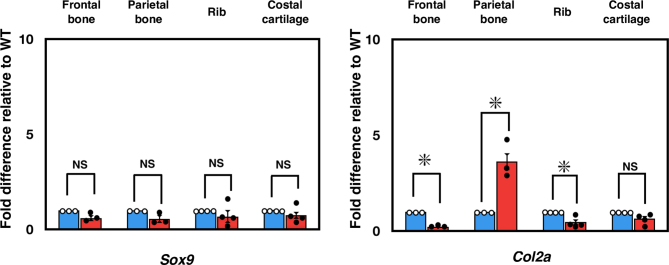
Expression of mRNA for chondrogenesis.

To better understand the role of *Ids*-deficiency in osteoblastogenesis *in vivo*, we examined two osteoblastic marker genes, such as *Col1a* and *Runx2* by quantitative RT-PCR (Fig. 5A). Among many skeletal specimens, we particularly focused on the expression of these genes in frontal bone and parietal bone as well as rib and costal cartilage, because the latter two are known to be actively involved in endochondral ossification. As we hypothesized, the expression of *Col1a* and *Runx2* was specifically enhanced in parietal bone, but not in frontal bone or rib and costal cartilage (Fig. 5A). In contrast, when we examined the changes in expressing chondrogenesis-related genes, such as *Sox9* and *Col2*, these remained unaltered (Supplementary Fig. S7). Apart from gene expression, when we investigated cell proliferation using the Ki-67 antigen, a widely accepted marker for this purpose, we observed normal Ki-67 expression in the frontal bone and parietal bone of *Ids*-deficient mice through immunohistochemistry. Ki-67 expression was increased in osteoblasts but not in osteocytes in these mice(Fig. 5B). These results indicated that an enhanced osteoblastogenesis is specifically enhanced in the parietal bone. In summary, we conducted parietal bone measurements with a digital caliper, and assessed the thickness of osteoblast tissue with H&E staining. The skeletal phenotype of Ids-deficient mice includes a noticeable increase in the size of the calvarial bone, both in neonatal and young adult mice.

### 3.4 Enhanced differentiation of *Ids*-deficient osteoblast

**Fig. 6:**
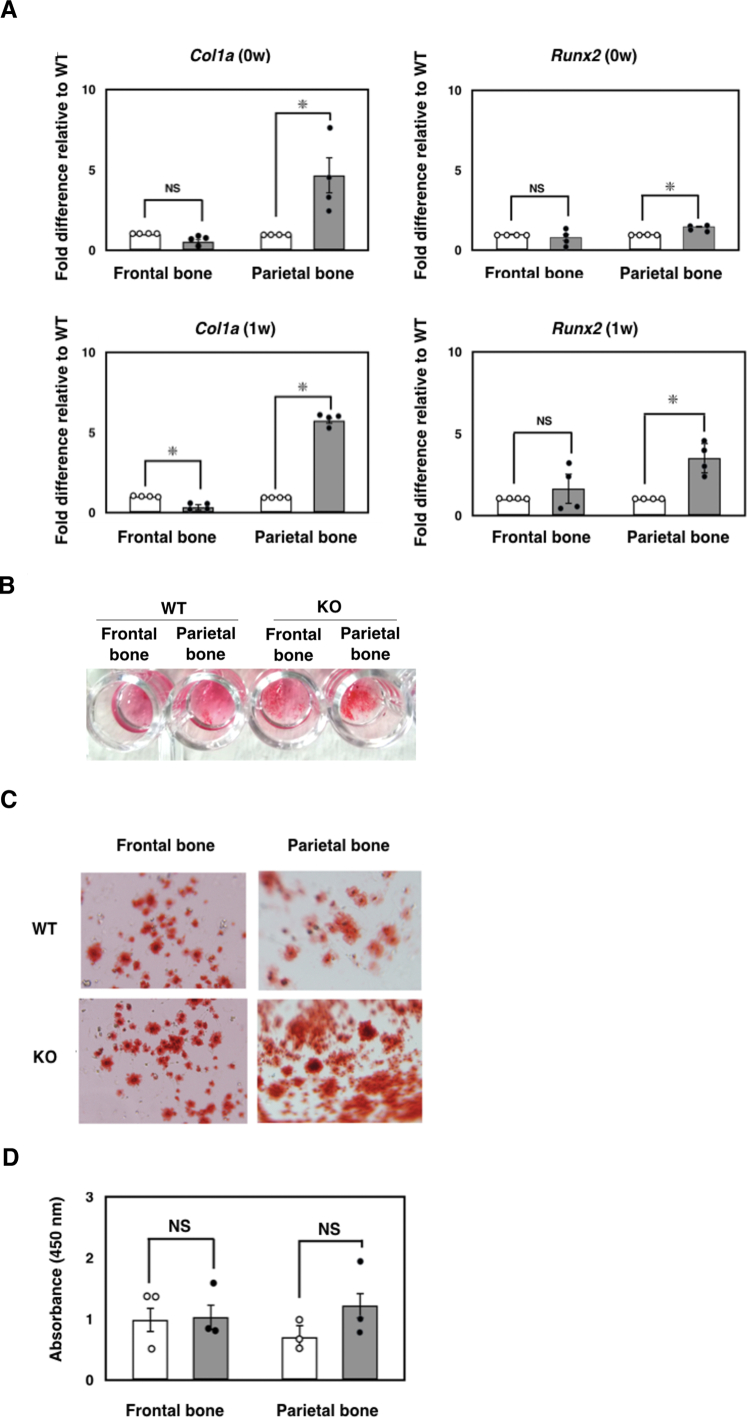
Enhanced differentiation of *Ids*-deficient osteoblast *in vitro*. (A) Enhanced expression of osteoblastic markers examined using quantitative RT-PCR. Total RNA was isolated and reverse transcribed as described. An aliquot (0.1 ng) of cDNA was used as the template. Expression of *Col1a* and *Runx2* was measured based on the expression of *Gapdh* as a control. An asterisk (*) indicates a significant difference between two groups (P < 0.05). (B, C) Enhanced calcification of *Ids*-deficient osteoblast prepared from frontal bone and parietal bone. Osteoblast was fixed with 4% paraformamide for 30 min followed by treatment with Alizarin red for a further 30 min. Accumulation of crystalized Alizarin red was photographed using a digital camera (B) or a digital microscope (C). (D) Unaltered cell proliferation of frontal bone and parietal bone in *Ids*-deficient osteoblast. We utilized a cell proliferation assay called CCK-8, where cell proliferation was measured through absorbance at 450 nm using a spectrophotometer. Open and gray bars represent the mean value of wild-type control osteoblast and *Ids*-deficient osteoblast, respectively.

Based on this evidence, we hypothesized whether osteoblastogenesis might be enhanced in the *Ids*-deficient osteoblast, particularly in parietal bone. To test this idea, we first prepared the osteoblast from the frontal bone and parietal bone from wild-type controls and *Ids*-deficient mice by an established collagenase-mediated isolation protocol, and then further examined the expression of osteoblast-specific marker genes. As expected, we found a marked elevation of *Col1a* and a marginal expression of *Runx2* in *Ids*-deficient parietal bone, but not the frontal bone in the primary culture immediately after the isolation (Fig. 6A, Top). Intriguingly, the expression of osteoblastogenesis markers was further enhanced in the *Ids*-deficient parietal bone. In sharp contrast, the expression of *Runx2* remained unaltered in the frontal bone (Fig. 6A, Bottom). To further examine the alteration of function in an *Ids*-deficient osteoblast, we examined the ability of calcification as matured osteoblast by crystallization of Alizarin red as the measure. Consistent with an enhanced gene expression of *Col1a* and *Runx2* in the *Ids*-deficient osteoblast, these cells also exhibited a marked crystal formation of Alizarin red (Fig. 6B and C). In sharp contrast, although there was a weak trend in the increase of cell proliferation of the parietal bone in *Ids*-deficient cells, there was no significant difference between the proliferation of the osteoblast of the wild-type control and *Ids*-deficient mice (Fig. 6D), demonstrating that an enhanced growth of calvarial bone, in particular the parietal bone, was due to an enhanced differentiation to osteoblast from progenitor cells.

### 3.5 Enzyme replacement suppressed GAG accumulation in *Ids*-deficient osteoblast isolated from parietal bone

**Fig. 7:**
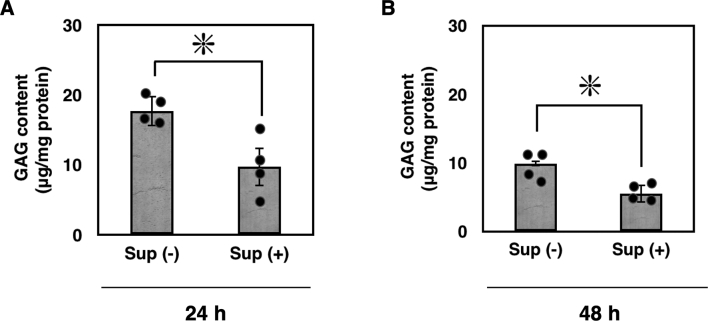
Enzyme replacement suppressed GAG accumulation in *Ids*-deficient osteoblast isolated from parietal bone. (A) An aliquot of IDS-enriched culture medium was added to the osteoblast of *Ids*-deficient cells (gray bar) at 0.3 μg/mL in Sup(+), and these cells were cultured for an additional 24 h. Subsequently, the concentration of GAGs in the cell lysate was determined using a 1,9-dimethylmethylene blue-mediated colorimetric assay kit. The GAG content was expressed as μg/mg protein. For control, no IDS enzyme was added in Sup(−). (B) An aliquot of IDS-enriched culture medium was added to the osteoblast in *Ids*-deficient cells (gray bar), and these cells were cultured for an additional 48 h. Sup, supernatant.

We established modified cell lines derived from baby hamster kidney cells (BHK-21 cells) that consistently produce human IDS. In the culture supernatant, we measured IDS activity at 2 μmol/h/L [33]. We then examined whether supplementing this exogenous IDS enzyme could correct the Ids-deficient phenotypes. When we added an exogenous IDS enzyme and incubated them for 24 h at 37 °C, as expected, this increased GAG concentration was reduced (Fig. 7A). Consistently, when we extended the incubation period with IDS enzyme and osteoblasts up to 48 h, demonstrating a reduction in the concentration of GAG in these Ids-deficient osteoblasts (Fig. 7B).

## 4 Discussion

In this study, we reported the establishment of a novel murine model of MPS II, and further provided evidence that our animal model showed a similar phenotype of previous models at the biochemical and pathological basis. Furthermore, this model displayed an enhanced osteoblastic development in cranial bone. Since cranial bone consists of neural crest, cell-derived frontal bone and paraxial mesoderm-derived parietal bone, we aimed to differentiate which cell type was more severely affected. Based on our data, a lack of *Ids* led to a more profound effect in the parietal bone, as judged by an enhanced thickening of parietal bone, expression of LSD-related genes, and accumulation of GAG. In sharp contrast, the frontal bone showed a rather mild effect of *Ids* deficiency in osteoblastic differentiation by gene expression analysis using quantitative RT-PCR. Finally, both proliferation of osteoblast isolated from the parietal bone and frontal bone remained unaltered regardless of genotype, demonstrating that a deficiency of *Ids* had no impact on osteoblastic proliferation.

An enlarged head circumference is a major skeletal manifestation that several studies reported [[34], [35], [36], [37]]. Among them, a study of the Hunter Outcome Survey—the largest data repository of MPS II-affected individuals—reported that, at least in part, the average head circumference of patients aged between 2 and 12 was larger than that of the control subjects [34]. Based on these clinical data, it is reasonable to hypothesize that membranous ossification is negatively associated with IDS enzyme, although the precise mechanism remains currently uncharacterized. In contrast, the height of MPS II-affected individuals aged above 6 years old is lower than the controls. This demonstrates that endochondral ossification of MPS II-affected individuals was attenuated. Interestingly, skull thickening is also noted in the small percentage of MPS II-affected individuals. Consistent with these clinical data, our observation in *Ids*-deficient mice showed an enlarged head as well as skull thickening (Fig. 3E-G). Thus, a further study will use our animal model to elucidate the precise molecular mechanism. Finally, the severity of bone deformity is dissociated from the cognitive involvement.

Intramembranous ossification is a type of osteogenesis that plays a key role in developing the calvarial bone, clavicle bone, and a part of the mandible bone [14,15]. As the mechanism of differentiation, mesenchymal cells are known to differentiate into osteoblasts without chondrogenesis, followed by forming the osteocyte. Intramembranous ossification has been considered a primitive mechanism of bone formation, typically occurring through endochondral ossification, which is a widely observed mechanism of the formation of long bones. Several studies have suggested that the osteoblast from the frontal bone differentiates faster than in the parietal bone [30,38,39]. Thus, we initially hypothesized whether the osteoblast derived from the frontal bone in *Ids*-deficient mice exhibits an enhanced differentiation. In fact, we found an enhanced differentiation of the osteoblast derived from the parietal bone rather than the frontal bone, suggesting that this phenotype appeared to be dissociated from gene regulation in the origin of cells, such as neural crest cells for the frontal bone and paraxial mesoderm for the parietal bone. Deficiency of Axin, a negative regulator of Wnt signaling, in parietal bone increases the osteoblastic differentiation as seen in our model, raising the possibility that *Ids* deficiency could enhance Wnt signaling in these cells [30]. Apparently, the development of bone is affected by multiple factors, such as the function and quantity of osteoblast, osteoclast, local fibroblast, hematopoietic cells, and others [14,15]. A secondary effect— the paracrine/endocrine effect of cytokine signaling, such as Wnt [40], BMP [41], and FGF [42], respectively—might be involved, as well. In addition, transcription factors that regulate bone patterning is another important factor [43,44]. These molecular mechanisms are presently under investigation with the aim of achieving a better understanding at the single-cell resolution, particularly within certain calvarial regions, such as the coronal suture [45]. Expansion of a similar technique to better understand the molecular mechanism with spacio-temporal resolution will further assist the development of novel therapeutic strategy for bone abnormality in MPS II.

Bone deformity is a representative manifestation of mucopolysaccharidoses with an increasing dermatan sulfate as the biomarker. In other words, mucopolysaccharidosis types I, II, VI, and VII are closely associated with this phenotype. Currently, enzyme replacement therapy is available and is generally effective for visceral disorders such as splenomegaly and hepatomegaly. In contrast, this treatment is not obviously efficient for treating bone deformities, particularly once it is established. Apart from this clinical observation, the osteoblast's manifestation was corrected by enzyme replacement in the MPS VI model *in vitro* [46]. We also discovered the positive effect of GAG normalization in *Ids*-deficient osteoblasts under similar experimental settings (Fig. 7). Consistent with these cellular observations, a recent study reported that an improvement of bone deformity may be possible using a lentiviral vector-mediated strategy [25]. Although a detailed mechanism may be established, it was argued that an *Ids*-transduced osteoclast appeared to provide active IDS enzyme to the osteoblast. Although the effectiveness of currently available therapy for correcting bone phenotype in animal models is not consistent, the result of this animal study raises the possibility that increasing expression levels of therapeutic enzymes by genetic method leads to an increasing opportunity of correcting established bone disorders.

In summary, we described a novel murine model for MPS II and have provided evidence that this disease model recapitulated previously established phenotype at biochemical basis. We also revealed an enhanced osteoblastic differentiation in this model, raising the possibility that the development of a therapeutic method for skeletal manifestations might be accelerated. Further study involving the interaction between the osteoblast and other cells that might affect the patterning, differentiation, and proliferation of osteoblastic progenitor cells needs to be performed in order to better understand the whole picture of the physiological role that *Ids* has on osteoblastic differentiation in the body.

The following are the supplementary data related to this article.

Supplementary Information Enhanced osteoblastic differentiation of parietal bone in a novel murine model of mucopolysaccharidosis type II Supplementary material 1

## Author contributions

NY performed the experiment and drafted. MOh performed the enzyme assay using LC-MS/MS experiment. ST contributed the generation of mice. AO, MOn, MN, and TO provided scientific input. NY and RM designed the project. RM wrote the manuscript.

## Author statement

In accordance with the submission requirements of Molecular Genetics and Metabolism Reports, we provide the following Author Statement for our manuscript titled “Enhanced osteoblastic differentiation of parietal bone in a novel murine model of mucopolysaccharidosis type II”.

## Declaration of Competing Interest

The authors declare no competing financial interests.

## Data Availability

Data will be made available on request.
