# Supplementary material for: Enhanced osteoblastic differentiation of parietal bone in a novel murine model of mucopolysaccharidosis type II
Source: Mol Genet Metab Rep. 2023 Nov 11;37:101021. doi: 10.1016/j.ymgmr.2023.101021 (PMC10694741; doi:10.1016/j.ymgmr.2023.101021)
Supplement: Supplementary Information — Enhanced osteoblastic differentiation of parietal bone in a novel murine model of mucopolysaccharidosis type II [file mmc8.docx]

**Supplementary Information**

**Enhanced osteoblastic differentiation of parietal bone in a novel murine model of mucopolysaccharidosis type II**

Narutoshi Yamazaki^1,2^*, Mari Ohira^1^, Shuji Takada^3^, Akira Ohtake^2,4^, Masafumi Onodera^5^, Mahito Nakanishi^6^, Torayuki Okuyama^1,2^ & Ryuichi Mashima^1^

^1^Department of Clinical Laboratory Medicine, National Center for Child Health and Development, 2-10-1 Okura, Setagaya-ku, Tokyo 157-8535, Japan,

^2^Department of Clinical Genomics, Faculty of Medicine, Saitama Medical University, Saitama 350-0495, Japan, ^3^Department of Systems BioMedicine, National Research Institute for Child Health and Development, 2-10-1 Okura, Setagaya-ku, Tokyo 157-8535, Japan, ^4^Center for Intractable Diseases, Saitama Medical University Hospital, Saitama 350-0495, Japan, ^5^Department of Human Genetics, National Research Institute for Child Health and Development, Tokyo, Japan, ^6^TOKIWA-Bio Inc., 2-1-6 Sengen, Tsukuba-city, Ibaraki 305-0047, Japan.

*Correspondence: Narutoshi Yamazaki, MD.

Email: yamazaki-n@ncchd.go.jp; FAX: +81-3-3417-2238.

**Supplementary Methods**

**Genotyping PCR and DNA sequencing.** A 3-mm tip of mouse tail was excised and collected into a clean DNase-free PCR tube. Genome DNA was prepared using Extract-N-Amp™ PCR kit (Sigma-Aldrich, Saint Louis, MO) according to manufacture's instruction. After dilution of the sample, PCR-mediated ampliﬁcation was performed using ReadyMix™ Taq PCR Reaction Mix (Sigma-Aldrich) and ProFlex™ PCR System (Applied Biosystems, Waltham, MA). For further genome analysis, the PCR product from WT and KO mice was separated form 3% agarose gel by electrophoresis, excised from gel, and isolated using a Wizard genomic DNA purification kit (Promega, Madison, WI). Then, the reaction mixture was treated with an ExoSAP-IT Express PCR Product Cleanup kit (Applied Biosystems) and labeled with a BigDye Terminator v3.1 Cycle Sequencing Kit (Applied Biosystems). Finally, DNA sequencing was performed using a Genetic Analyzer 3130xl (Thermo Fisher Scientiﬁc, Waltham, MA).

**RNA isolation, cDNA synthesis, and quantitative reverse-transcription PCR.** Total RNA was isolated from the tissues and cells by the Sepasol®-RNA I Super G (Nacalai Tesque, Kyoto, Japan). The concentration of the isolated RNA was determined spectrophotometrically using a NanoDrop2000 (Thermo Fisher Scientiﬁc, Waltham, MA). The cDNA was synthesized using a GeneAce cDNA synthesis Kit (Nippon gene, Tokyo, Japan) at 42℃ in the presence of oligo (dT)15 primer (Takara Bio, Tokyo, Japan) according to the manufacturer's instruction. Then, PCR ampliﬁcation was carried out with THUNDERBIRD® SYBR qPCR Mix (Toyobo, Tokyo, Japan) using cDNA as a template. PCR primers were described in Supplementary Table S1. The level of expression was normalized to *Gapdh* and presented as fold increase.

**Tissue harvesting.** Frontal osteoblasts and parietal osteoblasts were derived from skull of mice at postnatal day (P) 0-2. Then, pericranium and dura mater were dissected off from the skull and the cranial suture were also removed. Visceral tissues were dissected and weighted.

**Statistical analysis.** Statistical difference between mean values are examined by Student’s test. P value <0.05 was considered statistically significant. Data were expressed as mean ± standard error of mean.

Supplementary Table S1 List of primers used in this study.

| Genes | Direction | Sequence (5'-3') |
| --- | --- | --- |
| *Gapdh* | Forward | TGCACCACCAACTGCTTAG |
|  | Reverse | GGATGCAGGGATGATGTTC |
| *Col1a* | Forward | GCTCCTCTTAGGGGCCACT |
|  | Reverse | CCACGTCTCACCATTGGGG |
| *Runx2* | Forward | CCCAACCGAGTCATTTAAGGCT |
|  | Reverse | GCTCACGTCGCTCATCTTG |
| *Col2a* | Forward | CACCAAATTCCTGTTCAGCC |
|  | Reverse | TGCACGAAACACACTGGTAAG |
| *Sox9* | Forward | AGCTCACCAGACCCTGAGAA |
|  | Reverse | TCCCCAGCAATCGTTACCTTC |
| *Hexb* | Forward | CATCGACCACAGTCCCAATTC |
|  | Reverse | CCAAAAACATAGTTGTAATATCGCC |
| *Idua* | Forward | TAGGTGCCGTACCTCACAGT |
|  | Reverse | CCAGGTGACTTCCTGGCTG |
| *Gusb* | Forward | TCTGAAACCCGCCGCATATT |
|  | Reverse | CGTTGCTCACAAAGGTCACG |
| *Ids* exon 1-2 | Forward | GCTTCAGCTTGTTGCTAGGC |
|  | Reverse | GGGCGGAGATCATCCACAAT |
| *Ids* exon 5-6 | Forward | AAGCCGCATATCCCCTTCAG |
|  | Reverse | GGAACATGGGGATCAGGAGC |
| *Ids* exon 7-8 | Forward | TCGTCTAGCCCACAACACAA |
|  | Reverse | TACTTGGCCCATTCCTCCGTG |

**Supplementary Figure Legend**

**Supplementary Figure S1 Genotyping of *Ids*-deficient mice.** (A) PCR amplification of genomic DNA isolated from mouse tail biopsies. A single band corresponding to the amplicon of wild-type *Ids* allele with 370-bp and that of *Ids*-deficient allele with 220-bp were shown. Sequence of primers was summarized in Supplementary Table S1. (B) **PCR-mediated sex determination of mice.** The size of amplicon for *Ube-X* for female was 217 bp, whereas that for *Ube-Y* for male was 198 bp.

**Supplementary Figure S2 Ratio of mutant animals as determined by genotyping.** The ratio of wild-type controls to *Ids*-deficient mice in male (Left, *n* = 121), heterozygotes to *Ids*-deficient homozygotes in female (Center, *n* = 116), and the sum of both (Right, *n* = 237) was presented.

**Supplementary Figure S3 Nucleotide sequence and expression of matured mRNA in *Ids*-deficient mice used in this study.** (a) PCR product of cDNA prepared from *Ids*-deficient sample. Representative image of agarose gel electrophoresis was shown. Under this experimental condition, an accumulation of a single band with 650-bp (wild-type) and 470-bp (*Ids*-deficient) was detected. The sequence of primers was described in Supplementary Table S1. (b) Nucleotide sequence of mRNA from wild-type control and *Ids*-deficient mice. Electropherogram of nucleotide derived from cDNA from wild-type control tissue and *Ids*-deficient tissue was shown. Highlighted sequence in yellow in color indicated the nucleotide sequence of *Ids* gene in wild-type control (Top) which is missing in *Ids*-deficient cDNA (Bottom). (c) Schematic representation of detailed nucleotide sequence in *Ids*-deficient mice. In *Ids*-deficient mice, all nucleotides of exon 3 (178 bp) were missing. (d) Expression of *Ids* mRNA using different set of primers. Total RNA was prepared from wild-type control tissue and *Ids*-deficient tissue and reverse-transcribed. Expression of *Ids* mRNA was examined using primers that selectively amplifies exons 1-2, exons 5-6, and exons 7-8, respectively. Data were expressed as relative expression using *Gapdh* as a standard. Note that the expression of mutant *Ids* mRNA was reduced at approximately 25% in all combination of primer set.

**Supplementary Figure S4.** The weight of organ was examined at 13 weeks of age (wild-type, *n* = 3; *Ids*-deficient, *n* = 3).

**Supplementary Figure S5.** Whole-mount Alizarin Red S/Alcian Blue staining. P1 mouse was sacrificed and fixed with 10% formalin for 2 days. Then, the body was trimmed by removing skin, muscle, and internal organs, and then further treated with a mixture of enzyme Polident (GSK Japan, Tokyo, Japan) at room temperature for 1 week. Next, the samples were stained with 3% Alcian blue (Nacalai Tesque) in 0.5% potassium hydroxide for 3 minutes, treated with digestion enzyme, stained with 1% Alizarin red (Sigma-Aldrich) for 30 minutes, and treated with a graded series of glycerin. Finally, the specimen was photographed using a digital camera. The arrow in wild-type animal indicated the cartilage of the parietal bone (blue). Note that the same position *Ids*-deficient mice showed advanced ossification (red).

**Supplementary Figure S6 Masson’s trichrome staining of cranial bone.** A formalin-fixed tissue was embedded in paraffin and a 7-μm section was stained. Collagen was selectively stained in blue in this staining. Scale bar = 100 μm.

**Supplementary Figure S7 Expression of mRNA for chondrogenesis.** The expression of *Sox9* and *Col2a* in frontal bone, parietal bone, rib, and costal cartilage in *Ids*-deficient mice was examined using quantitative RT-PCR. *Gapdh* was used as a control.
